# Chandipura Virus Causing Large Viral Encephalitis Outbreaks in India

**DOI:** 10.3390/pathogens13121110

**Published:** 2024-12-16

**Authors:** Morgan E. Brisse, Hinh Ly

**Affiliations:** 1 Viral Immunity and Pathogenesis Unit, Laboratory of Viral Diseases, National Institute of Allergy and Infectious Diseases (NIAID), National Institutes of Health (NIH), Bethesda, MD 20892, USA; 2 Department of Veterinary & Biomedical Sciences, College of Veterinary Medicine, University of Minnesota, Twin Cities, MN 55108, USA

Chandipura virus (CHPV) is a negative-, single-stranded RNA virus belonging to the family *Rhabdoviridae* [1]. CHPV is transmitted by the sand fly species *Phlebotomus* sp. and *Sergentomyia* sp. as well as by the mosquito *Aedes aegypti*. Human-to-human transmissions of CHPV have not been documented. CHPV infection can lead to pediatric encephalitis (brain inflammation in children) [2] that can result in the mortality rate ranging from 44% to 75% [3,4]. Other symptoms of severe CHPV infection include high-grade fever, vomiting, convulsions, and coma, which can culminate in death within 2 days of hospitalization. The most recent outbreak of CHPV took place in June–August 2024 across several districts in Gujarat, India [5,6], where at least 78 cases of acute encephalitis syndrome (AES) were identified in children under 15 years old. According to a press release on 20 July 2024 by the Ministry of Health and Welfare of the Government of India, most of the cases (75) were from 21 Gujarati districts and corporations, two cases were from Rajasthan, and one case was from Madhya Pradesh, resulting in 28 deaths, among which children were most impacted [7]. Nine of 76 patients’ samples were sent to the National Institute of Virology in Pune, India, and tested positive for CHPV. All of those nine CHPV-positive patients, along with five other related deaths due to CHPV infections were from Gujarat, India [7]. A recent report by the World Health Organization (WHO) listed the total number of confirmed and suspected CHPV cases at 245 with a case fatality rate of 33% [8].

CHPV was first discovered in 1965 during an outbreak in the Chandipura village of the Nagpur district in Maharashtra state, India, and hence it was named the Chandipura virus [2]. CHPV was initially isolated from two cases in adult humans presenting with fever and joint pain [2]. CHPV has since caused several large outbreaks throughout India, as shown in Figure 1. Rao and colleagues identified CHPV as the causative infectious agent of a large encephalitis outbreak in Andhra Pradesh, India, from June to September 2003 that led to a very high rate of fatality (~55%) [3]. A total of 7 CHPV outbreaks have occurred since 2003 (including the 2024 outbreak), which tended to peak with the monsoon season and had case fatality rates between 28 and 79 percent [1,3,9,10,11,12,13,14] (Table 1). CHPV has also been suspected as an etiological pathogen in historical outbreaks of high mortality that occurred prior to the availability of genetic sequencing, which were previously attributed to other endemic infectious diseases such as dengue, chikungunya, measles, and Japanese encephalitis virus [9,15,16,17]. CHPV likely causes low levels of human cases in between outbreaks, with one study detecting CHPV RNA in 3/278 cases of acute encephalitis syndrome (AES) in India from 2018 to 2020 [18].

Although CHPV has been repeatedly found in human patients in India, it has also been isolated from arthropods (e.g., sand flies) in Senegal, Kenya, and Nigeria on the African continent. Macaques (monkeys) from Sri Lanka have also been identified as being seropositive (containing anti-CHPV antibodies in serum). Together, these observations suggest that the geographical distribution of CHPV may be wider than previously thought and might include regions that have not yet had significant outbreaks in humans [4,19,20]. This could be further accentuated by the rapid expansion of habitable environments for the *Aedes aegypti* mosquito [21] due to climate change, thereby leading to the expansion of CHPV from the Indian and African subcontinents to other parts of the world. Therefore, continuing surveillance studies for CHPV prevalence must be prioritized to prevent future outbreaks. Additionally, studies of CHPV transmission, pathogenicity, prophylactics, and therapeutic treatments are critical for the development of better forms of treatment against this deadly vector-borne infection, as there are no current treatments for severe CHPV infection except for supportive care.

Recent studies have shown ribavirin and related compounds, such as favipiravir (T705), as well as other novel antiviral compounds, such as vesiculopoins, could suppress CHPV replication in vitro [22,23,24]. The inhibitory effect of favipiravir (T705) toward CHPV replication has also recently been tested in vivo [23]. Using a lethal mouse model consisting of 10-day-old CD1 mice intraperitoneally inoculated with 10,000 PFU (plaque-forming units) of CHPV, favipiravir (T705) treatments orally at 100 mg/kg/day and 300 mg/kg/day were found to increase the survival rates of the animals by 75% and 100%, respectively. Dosing with 300 mg/kg/day appeared to clear the virus in the serum, spleen, and brain of the animals on days 4, 8, and 21 of treatments. By contrast, untreated mice had high viral titers at 4 days post-infection and succumbed to the infection by day 6 post-infection. Similar results were also noted in another study using SCID mice, further supporting the potential use of favipiravir (T705) against CHPV infection [25]. It must be emphasized that the potential efficacy and safety of favipiravir (T705) in humans must be demonstrated in clinical trials prior to its widespread clinical use. Future research into CHPV transmission, pathogenicity, prophylactics, and additional therapeutic strategies is essential as CHPV remains a constant threat to public health.

## Figures and Tables

**Figure 1:**
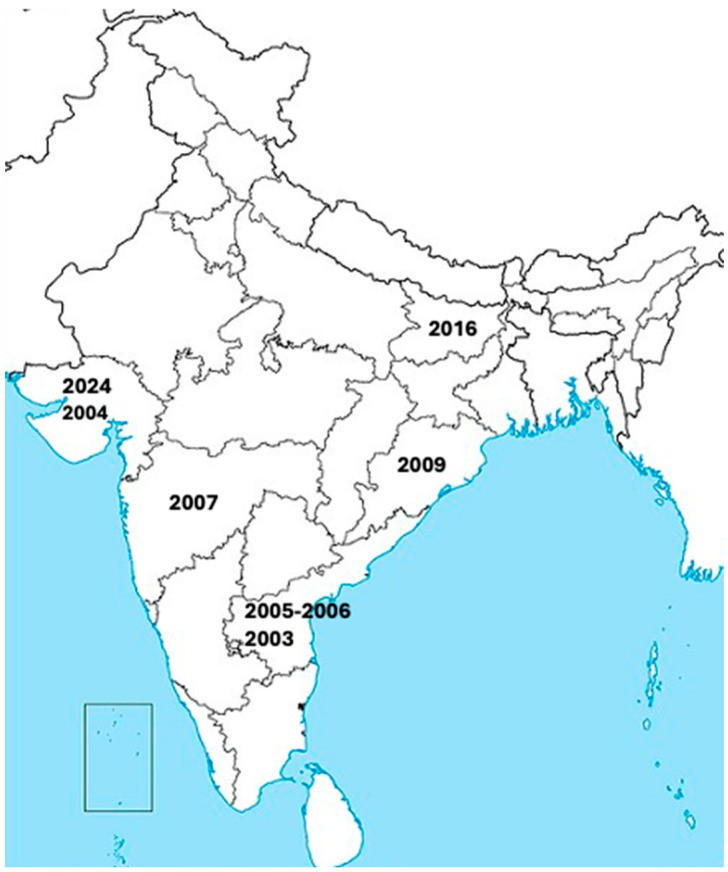
Locations of several known large outbreaks of human CHPV infections throughout India from 2003 to 2024.

**Table 1:** Summary of recorded CHPV outbreaks. A total of 7 CHPV outbreaks have occurred since 2003, which tended to peak with the monsoon season and had case fatality rates between 28 and 79 percent [1,3,9,10,11,12,13,14]. The table includes the date(s) of outbreaks, regions affected, total number of acute encephalitis syndrome (AES) cases, the age of affected patients, and the case fatality rate (CFR). Historical data (prior to 2024) adapted from reference 14.

Date *	Regions #	AES Cases ^	Ages Affected ~	CFR ”
2024	Gujurat	245	Under 15 years	33%
2016	Bihar	24	1-15 years	20.8%
2009	Odisha	21	Under 10 years to over 18 years	28.6%
2007	Maharashtra	78	Under 15 years	43.6%
2005–2006	Andhra Pradesh	52	Under 15 years	54.4%
2004	Gujurat	20	2 to 16 years	78.3%
2003	Andhra Pradesh	55	2.5 months to 15 years	54.9%

* Date in years when AES outbreak was reported. # Indian states with reported cases in each outbreak. ^ Total number of AES cases (confirmed and suspected cases of CHPV infection) in each outbreak. ~ The age range of AES patients in each outbreak. ” The reported case fatality rate (CFR) for each outbreak calculated from total AES cases.

## Data Availability

No primary data are included in this article.
